# Joint Deep Reinforcement Learning and Unsupervised Learning for Channel Selection and Power Control in D2D Networks

**DOI:** 10.3390/e24121722

**Published:** 2022-11-24

**Authors:** Ming Sun, Yanhui Jin, Shumei Wang, Erzhuang Mei

**Affiliations:** 1 College of Computer and Control Engineering, Qiqihar University, Qiqihar 161006, China; 2 School of Computer and Information Engineering, Harbin University of Commerce, Harbin 150028, China

**Keywords:** device-to-device, channel selection, power control, deep reinforcement learning, unsupervised learning

## Abstract

Device-to-device (D2D) technology enables direct communication between devices, which can effectively solve the problem of insufficient spectrum resources in 5G communication technology. Since the channels are shared among multiple D2D user pairs, it may lead to serious interference between D2D user pairs. In order to reduce interference, effectively increase network capacity, and improve wireless spectrum utilization, this paper proposed a distributed resource allocation algorithm with the joint of a deep Q network (DQN) and an unsupervised learning network. Firstly, a DQN algorithm was constructed to solve the channel allocation in the dynamic and unknown environment in a distributed manner. Then, a deep power control neural network with the unsupervised learning strategy was constructed to output an optimized channel power control scheme to maximize the spectrum transmit sum-rate through the corresponding constraint processing. As opposed to traditional centralized approaches that require the collection of instantaneous global network information, the algorithm proposed in this paper used each transmitter as a learning agent to make channel selection and power control through a small amount of state information collected locally. The simulation results showed that the proposed algorithm was more effective in increasing the convergence speed and maximizing the transmit sum-rate than other traditional centralized and distributed algorithms.

## 1. Introduction

With the development of wireless communication technology and the increasing number of mobile devices, a large amount of data traffic needs to be transmitted over wireless networks, due to the increase in user demands for high data transmission services [1,2,3,4,5]. Operators often deploy more base stations (BSs) in multiple frequency bands to relieve mobile congestion, but this also leads to significant cost expenditures. The emerging device-to-device (D2D) technology enables direct communication between devices and offloads the heavy mobile traffic from BSs at a low cost [6]. In the existing technology, there are mainly two multiplexing modes, namely, underlay mode and overlay mode. In underlay mode, DUEs (Device User Equipment) and CUs (Cellular Users) are allowed to transmit data at the same time, which unavoidably generates interference between DUEs and CUs. However, this paper focuses on the overlay mode. In this mode, a part of the spectrum resources can be used by D2D pairs alone, which eliminates the interference caused by partitioning orthogonal spectrum resources for DUEs and CUs [7]. In most scenarios, the number of available channels in a D2D network is usually much smaller than that of DUEs. As a result, multiple DUEs have to share the same channel, resulting in serious co-channel interference among DUEs.

In order to reduce the co-channel interference between D2D users, the problem of the channel selection and the power allocation has been widely studied. According to the differences of the required channel state information (CSI), models to solve the mentioned problem above can be categorized into centralized [8,9,10,11,12,13] and distributed [14,15] models. In general, centralized models require global CSI, while distributed models require only local CSI.

In centralized models, the iterative algorithms, such as the fractional programming (FP) [8] and the weighted minimum mean square error (WMMSE) [9], are known to be the most advanced optimization methods. Both algorithms not only require the complete global CSI, but are also mathematically accurate and easy-to-handle models. Besides, deep learning techniques are also used in centralized models to solve the resource allocation problem in wireless networks due to low computational complexity [10,11,12,13]. However, it is a great challenge to obtain the global CSI in time for non-stationary wireless environments because of the following facts. For one thing, the channel state information varies quickly with non-stationary wireless environments. For another, large-scale information exchanges are needed for collecting the global CSI. As a result, the above aspects undoubtedly increase the difficulty in applying the centralized methods to non-stationary wireless environments. Therefore, most of the algorithms of centralized models are not applicable to practical large-scale network scenarios.

Distributed models can better account for the timeliness and relevance of information in real wireless networks. In distributed models, channel selection is usually time-dependent and varies according to some patterns based on channel state information. In the distributed model, Tan et al. [14] proposed to train a separate deep Q network that handled both channel selection and transmit power control for D2D user pairs. The main drawback of this algorithm was that the deep Q network (DQN) learning algorithm was not easily applied to problems that contained both discrete and continuous variables. To solve this problem, Nasir et al. [15] proposed to use a deep Q-network for the discrete channel allocation in the bottom layer, and a deep deterministic policy gradient (DDPG) for the continuous power allocation in the top layer. In the algorithm present in [15], the deterministic policy of the DDPG was highly dependent on the accuracy of the Q-value estimation. However, the neural network at the beginning of the training leads to a poor Q-value prediction, and the training with the DDPG strategy becomes less effective as the size of the neural network increases. The above two shortcomings reduce the efficiency of the deterministic strategy of the DDPG for action exploration, and thus the spectrum utilization cannot be improved efficiently and quickly with continuous power allocation from the DDPG.

Except for deep supervised learning and deep reinforcement learning, deep unsupervised learning is also widely used to solve the continuous power allocation problem for the physical layer of wireless networks. Since unsupervised learning techniques can directly model and analyze the data without labels, they can prevent inappropriate label sets from degrading the performance of neural networks. For example, researchers have used unsupervised learning techniques for power control solutions in D2D communication systems [16,17]; Liang et al. constructed integrated networks based on the idea of integrated learning and used unsupervised learning techniques for power control and for maximizing the sum-rate of multiple transceiver pairs in wireless networks [18]. As opposed to the deterministic strategy of the DDPG, which trains network parameters indirectly by estimating the Q value [15], the above deep unsupervised learning method trains network parameters directly by optimizing the gradient of the target, giving it higher network training efficiency.

Due to the scarce spectrum resources in the overlay mode, both the channel allocation and the power control are required to reduce the co-channel interference between DUEs. Inspired by the deep unsupervised learning approach, this paper proposes a distributed resource allocation algorithm, which combines the deep reinforcement learning and the deep unsupervised learning to investigate the channel selection and power control of multichannel D2D networks for maximizing the channel transmission rate. The proposed distributed algorithm in this paper includes a deep reinforcement learning-based deep neural network for channel allocation (DRLDNN-CA) and an unsupervised learning-based deep neural network for power control (ULDNN-PC). That is, the proposed DRLDNN-CA and the proposed ULDNN-PC can output the channel allocation scheme and the channel power, respectively. It should be noted that the proposed DRLDNN-CA uses the local information set collected by agents as input, while the ULDNN-PC uses a set of the local information determined by the output channel assignment scheme of the DRLDNN-CA as input. In addition, both the proposed DRLDNN-CA and the proposed ULDNN-PC were executed through distribution and trained in a centralized manner to maximize the transmit sum-rate.

The main innovation of this paper is to use deep reinforcement learning combined with unsupervised learning for channel allocation and power control in distributed D2D networks. First of all, the research on the interference suppression of D2D network technology is still insufficient. In addition, a centralized model requires global CSI, while a distributed model requires only local CSI. The distributed model can better consider the timeliness and relevance of information in real wireless networks. It is worth mentioning that in the distributed model, joint deep reinforcement learning and unsupervised learning have theoretical support and practical basis, but no academic research has been reported. In this paper, the deep reinforcement learning technology DRLDNN-CA was proposed for channel allocation, and the unsupervised learning technology ULDNN-PC was proposed for power control. Since unsupervised learning technology does not require labels, inappropriate labels can be prevented from affecting neural network performance, and unsupervised learning technology can directly model and analyze data. The results showed that DRLDNN-CA combined with ULDNN-PC technology was more effective than traditional centralized and distributed algorithms in improving convergence speed and in maximizing transmission and rate.

The rest of this paper is organized as follows. The system model of a multichannel D2D network is formulated in Section 2. In Section 3, the maximization problem of the transmit sum-rate for the multichannel D2D network is formulated. In Section 4, we first provide brief overviews for both reinforcement learning and unsupervised learning, and then describe the local state information available for distributed models, and finally propose the joint deep reinforcement learning and unsupervised learning framework for the computation of distributed resource allocation. Simulations and comparisons are presented in Section 5, and conclusions are shown in Section 6.

## 2. System Model and Related Works

### 2.1. System Model

**Figure 1:**
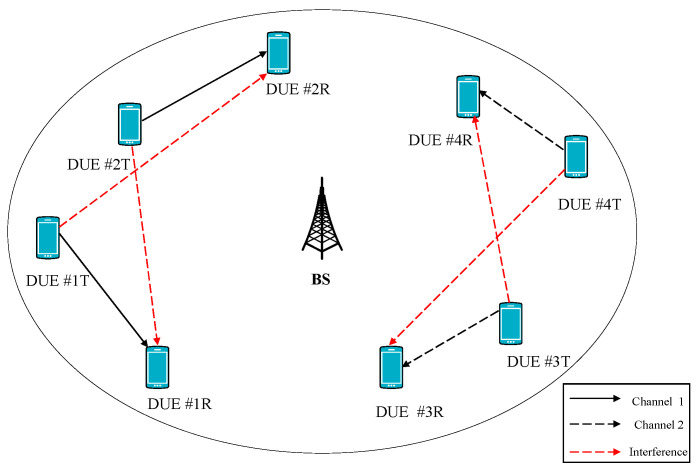
System model of a multichannel D2D network.

The system model of a multichannel D2D network with distributed control is shown in Figure 1.

In this paper, a multichannel D2D network with *n* links sharing *m* channels (*n* > *m*) is considered. The sets of links and channels are denoted as $n=\left\{1,\ldots ,N\right\}$ and $m=\left\{1,\ldots ,M\right\}$, respectively. As shown in Figure 1, link *n* consists of a D2D user transmitter (DUE T) and receiver (DUE R). Since DUE #1 and DUE #2 use channel 1 at the same time, the DUE #1T transmitter will cause interference to the DUE #2R receiver, and the DUE #2T transmitter will cause interference to the DUE #1R receiver. Similarly, since DUE 3 and DUE 4 use channel 2 at the same time, the DUE #3R receiver and the DUE #4R receiver will receive interference from the DUE #4T and DUE #3T, respectively. In this paper, we assume that the time slot system is fully synchronized and the length of a time slot is fixed, and that a link will select a channel at the beginning of each time slot.

In this paper, the channel model used is composed by large-scale fading and small-scale fading. Similar to [19], the small-scale fading between the transmitter and its receiver is assumed to be block fading, and the small-scale Rayleigh fading is described by the Jake’s model [20]. In this paper, $g^{\left(t\right)}$ and $g^{\left(t+1\right)}$ are used to represent the small-scale Rayleigh fading at *t* and *t* + 1, and the following equation is used to express the correlation between them [15].

(1) $$g^{\left(t+1\right)}=\rho g^{\left(t\right)}+\sqrt{1-\rho^{2}}e^{\left(t\right)}$$

where ρ∈[0,1] is used to represent the correlation between two consecutive decaying blocks, and both $g^{\left(0\right)}$ and $e^{\left(t\right)}$ are complex Gaussian random variables that obey the Rayleigh distribution.

In the following, we denote $\beta_{k\to n}$ as the large-scale path loss from transmitter *k* to receiver *n* over channels, denote $g_{k\to n,m}^{\left(t\right)}$ as the small-scale Rayleigh fading from transmitter *k* to the receiver on channel *m*. Thus, the channel gain between transmitter *k* and receiver *n* on channel *m* at time slot *t* can be expressed as follows.

(2) $$h_{k\to n,m}^{\left(t\right)}=\beta_{k\to n}|g_{k\to n,m}^{\left(t\right)}{|}^{2}$$

In addition, we define $\alpha_{n,m}^{\left(t\right)}$ to indicate whether link *n* selects channel *m* at time slot *t*. We use $\alpha_{n,m}^{\left(t\right)}=1$ to indicate that link *n* selects channel *m*; otherwise, $\alpha_{n,m}^{\left(t\right)}=0$. Finally, we denote $p_{n}^{\left(t\right)}$ as the transmit power of transmitter *n* at time slot *t*. Based on the above defined notations, the signal-to-noise ratio (SINR) on channel *m* at time slot *t* can be expressed as (3), since the channel resources are orthogonal.

(3) $$\gamma_{n,m}^{\left(t\right)}=\frac{\alpha_{n,m}^{\left(t\right)}h_{n\to n,m}^{\left(t\right)}p_{n}^{\left(t\right)}}{\sigma^{2}+\sum_{k\neq n}\alpha_{k,m}^{\left(t\right)}h_{k\to n,m}^{\left(t\right)}p_{k}^{\left(t\right)}}$$

where $\sigma^{2}$ is the additive white Gaussian noise power.

However, as the channel resources are not orthogonal, there exists interference among the adjacent channels. For this situation, the signal-to-noise ratio (SINR) on channel *m* at time slot *t* can be expressed as (4).

(4) $$\gamma_{n,m}^{\left(t\right)}=\frac{\alpha_{n,m}^{\left(t\right)}h_{n\to n,m}^{\left(t\right)}p_{n}^{\left(t\right)}}{\sigma^{2}+\sum_{k\neq n,\;\left|m*-m\right|\leq z}\alpha_{k,m*}^{\left(t\right)}h_{k\to n,m*}^{\left(t\right)}p_{k}^{\left(t\right)}}$$

where m* is the adjacent channel of *m*, and *z* is the maximum distance of adjacent channels.

The downlink transmit rate achieved by link *n* over channel *m* at time slot *t* is defined as follows:

(5) $$C_{n,m}^{\left(t\right)}=\log_{2}(1+\gamma_{n,m}^{\left(t\right)})$$

### 2.2. Related Works

In order to clearly make comparisons with our proposed algorithm, the state-of-the-art algorithms for the resource allocation of D2D networks are summarized in Table 1. It can be seen from Table 1 that the state-of-the-art algorithms extensively focus on the power control and/or the channel allocation in D2D networks, and various deep learning technologies, such as DQN [14,21,22,23,24], CNN [16,25], DDPG [15], DNN [16,17] and so on, are involved. For example, Tan et al. [14] proposed to use a deep Q network to handle both the channel selection and the power control for D2D user pairs in a distributed model; Yuan et al. [21] proposed to use a double deep Q network for both the channel selection and the power control for D2D user pairs in a distributed model; Nasir et al. [15] proposed to use a deep Q-network for the discrete channel allocation, and proposed to use a deep deterministic policy gradient (DDPG) for the continuous power allocation. Besides, unsupervised learning-based deep neural networks have been applied for the resource allocation for D2D wireless networks [16,17,18,26]. It is known that unsupervised learning techniques do not require labels in training deep neural networks, so they can prevent inappropriate label sets from degrading the performance of deep neural networks. When compared with the supervised learning strategy in [25], the unsupervised learning strategy can train various deep neural networks, such as CNN [16] and DNN, [17,18,26] more effectively and efficiently.

However, the algorithms mentioned above are still deficient for the resource allocation of D2D wireless networks. Firstly, because of the discretized action space, DQN has shortcomings in solving problems that contain both discrete and continuous variables. This means that DQN cannot reach the optimal solution in solving problems with continuous power variables. Secondly, the deterministic policy of DDPG is highly dependent on the accuracy of the Q-value estimation. Unfortunately, the critic neural network in DDPG at the beginning of the training easily leads to a poor Q-value prediction, which causes the training with the DDPG strategy to be less effective as the size of the neural network increases. Thirdly, research on the application of the unsupervised learning to aid allocations of both the continuous power variables and the discrete channel variables in D2D networks remain deficient.

## 3. Problem Formulation

To reduce the interference between channels and improve the overall spectrum efficiency, the transmit sum-rate of all the D2D pairs can be maximized by optimizing the channel and power allocation. Define $\alpha^{\left(t\right)}={\left[\alpha_{1,1}^{\left(t\right)},\alpha_{1,2}^{\left(t\right)},\ldots ,\alpha_{N,M}^{\left(t\right)}\right]}^{T}$ and $p^{\left(t\right)}={\left[p_{1}^{\left(t\right)},p_{2}^{\left(t\right)},\ldots ,p_{N}^{\left(t\right)}\right]}^{T}$ as the channel selection and channel power at time slot *t*, respectively. The maximization problem of the transmit sum-rate can be formulated as the following P1.

(6) $$\begin{array}{l} P1:\underset{\alpha^{\left(t\right)},p^{\left(t\right)}}{\max}\sum_{n=1}^{N}\sum_{m=1}^{M}C_{n,m}^{\left(t\right)}\\ s.t.{\;C}_{1}:0\leq p_{n}^{\left(t\right)}\leq P_{\max},\forall n\in \{1,\ldots ,N\}\\ {\;\;\;\;C}_{2}:\alpha_{n,m}^{\left(t\right)}\in \left\{0,1\right\},\forall n\in \{1,\ldots ,N\},\forall m\in \{1,\ldots ,M\}\\ {\;\;\;\;C}_{3}:\sum_{m}\alpha_{n,m}^{\left(t\right)}=1,\forall n\in \{1,\ldots ,N\} \end{array}$$

In problem P1, C_1_ indicates that the transmit power of a transmitter is non-negative and should be less than its maximum transmit power; C_2_ indicates whether link *n* selects channel *m* at time slot *t*; C_3_ indicates that each link can and only can select one channel.

The P1 problem is a non-convex mixed integer nonlinear programming problem, and it is challenging to solve the P1 problem directly. For *N* links and *M* channels, there will be $M^{N}$ channel allocation schemes for the optimization problem P1, which suggests that the number of channel schemes will grow exponentially as the number of links increases. In addition, the channel interference among D2D user pairs also makes the power control a complex optimization problem [27]. It is known that the traditional centralized optimization algorithms often require instantaneous global channel state information (CSI), and take many iterations to achieve convergent states [15]. In order to overcome the shortcomings of the traditional centralized optimization algorithms and reduce the computation complexity, we propose a joint deep reinforcement learning and unsupervised learning technique model, which can use a small amount of local information collected by agents and obtain near-optimal solutions by decomposing the channel allocation and the power allocation in P1.

## 4. Joint Deep Reinforcement Learning and Unsupervised Learning Framework

### 4.1. A Brief Overview of Reinforcement Learning

**Figure 2:**
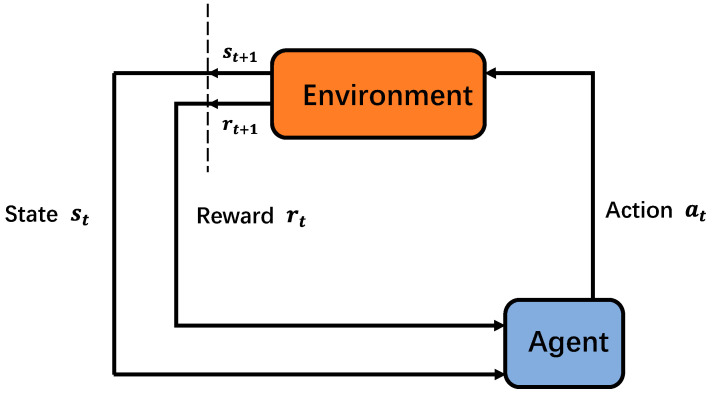
Basic structure of reinforcement learning.

Reinforcement learning is a process of continuous exploration and trial-and-error, where the agent learns strategies to maximize rewards by interacting with the environment. The basic structure of reinforcement learning is shown in Figure 2.

As shown in Figure 2, the interaction between the agent and the environment at time slot *t* is called the Markov Decision Process (MDP). All available actions made by the agent at time slot *t* comprise the action space A, and all the states that the agent observes in the environment make up the state space *S*. When the agent takes an action, the environment gives back a reward to evaluate the action at time slot *t*. The state of the agent at time slot *t* is assumed to be $s_{t}$, and the reward after the action a of $s_{t}$ is denoted as $f_{a(s_{t})}$. In addition, d(s) is used to represent a strategy, defined to be a mapping from states to actions, where d(s)∈A, s∈S. The ultimate goal is to find a strategy that maximizes the long-term cumulative discounted reward, which is denoted as follows.

(7) $$R^{t}=\sum_{t=0}^{\infty }\gamma^{t}f_{d(s_{t})}$$

where γ∈(0,1] represents the discount factor.

In the following, the Q-Learning algorithm [28] is introduced. In the Q-Learning algorithm, Q-values are the expected reward values obtained from the state-action (*s*, *a*) at time slot *t* under policy π, denoted as $Q^{\pi }(s,a)$.

(8) $$Q^{\pi }(s,a)=E\left[R^{t}\left|s_{t}\right.=s,a_{t}=a\right]$$

Let $Q^{\pi *}$ be the optimal Q-values $Q^{\pi *}$. Then the optimal $Q^{\pi *}$ satisfies the following Bellman equation.

(9) $$Q^{\pi *}(s,a)=Q^{\pi }(s,a)+\gamma \sum_{s_{t+1}\in S}P(s,s_{t+1})Q^{\pi *}(s_{t+1},\pi^{*}(s_{t+1}))$$

where $P(s,s_{t+1})$ is the transition probability from the state s to the next state $s_{t+1}$. Note that the Q-Learning algorithm updates Q-values by multiple iterations. Instead of always following some policy π, the agent takes action by using the ε-greedy method. That is, the agent takes an action by using the policy π with the probability of (1 − *ε*), while it takes a random action with the probability of ε. This enables the Q-learning algorithm to avoid local optimal solutions by exploring random actions.

However, Q-values in the Q-Learning algorithm are present in a state-action table, which results in that the Q-learning algorithm lacks generality and is not applicable to large discrete state spaces. To overcome this challenge, deep learning is combined with the Q-Learning algorithm to form the deep Q-Network algorithm (DQN) [3], which replaces the table with a deep neural network. The output of the DQN is denoted as Q(s,a;θ), where *θ* is denoted as the neural network parameters. Deep Q-learning is an off-policy learning method, which uses the experience replay method to store the past experience samples in the form of $M=(s_{t},a,r_{t+1},s_{t+1})$ into the experience pool. The advantage of setting up an experience pool is that it diversifies the data involved in training and makes it easy for the DQN to escape the local optimal solution.

The DQN algorithm adopts the “quasi-static target network” technique, which creates a target network with the parameter $\theta_{target}$. Then the loss function of the DQN network is defined as the mean-square Bellman error, as shown below.

(10) $$L(\theta )=E_{\left(s_{t},a,r_{t+1},s_{t+1}\right)}\left[{\left(y(s_{t+1},r_{t+1})-Q(s_{t},a;\theta )\right)}^{2}\right]$$

where $y(s_{t+1},r_{t+1})=r_{t+1}+\gamma \max_{a^{\prime }}Q_{target}(s_{t+1},a^{\prime };\theta_{target})$, and $Q_{target}$ is the Q-value of the target network.

In order to minimize the loss function (10), *M* batches are randomly selected from the experience pool, and stochastic gradient descent is performed to update θ.

### 4.2. A Brief Overview of Unsupervised Learning

Suren et al. [29] showed that the deep neural network using the unsupervised learning strategy could obtain better results on the resource allocation than using deep reinforcement learning strategy in a relatively stationary wireless network environment. When compared with the supervised learning strategy, the unsupervised learning strategy did not require supervision labels and could avoid decaying the performance of deep learning due to inappropriate supervision labels.

Currently, the unsupervised learning strategy has been used for power control solutions in D2D communication systems [16,17,18], where the negative minimum of the transmit sum-rate objective function was used as the loss function. However, the unsupervised learning strategy in [16,17,18] has shortcomings in the following two aspects. Firstly, the unsupervised learning strategy cannot be used for the computation of the distributed resource allocation with local state information. In addition, the unsupervised learning strategy can only be used for power control in single-channel D2D communication systems, while this is not possible in multichannel D2D communication systems. In this paper, we combine the deep reinforcement learning with the unsupervised learning strategy for power control in multichannel D2D communication systems under the framework of the distributed resource allocation.

### 4.3. Local State Information Available for Distributed Models 

Because centralized algorithms such as the FP algorithm [9] and WMMSE [10] algorithms are obliged to collect global CSI, they are suitable for stationary models without block fading, though not for large non-stationary models. In order for distributed execution to take place, multi-agent learning approaches [14,15] are often used, with each agent collecting the local state information, rather than just a single agent collecting the global state information, which can prevent similar drawbacks in centralized execution.

Similar to [15], the transmitter *n* is considered as the agent *n* in this paper. Besides, for collecting the local state information by each agent, both the interfering neighbor set and the interfered neighbor set are set for link *n*, $n\in \left\{1,\ldots ,N\right\}$. The interfering neighbor set of link *n* on the channel *m* is composed by those nearby transmitters, which interfere the receiver *n* on the channel *m* at the previous time slot t−1. The interfering neighbor set of link *n* on the channel *m* is denoted as $I_{n,m}^{\left(t\right)}$. In order to prioritize the transmitters that use channel *m*, the nearby transmitters are divided into two groups: those that occupy channel *m* at time slot t−1, and those that do not. Then, the interference intensity received at the receiver *n* (i.e., $h_{i\to n,m}^{\left(t-1\right)}$) is sorted by descending order in order to prioritize the transmitter neighbors that have the most serious interference impact on the receiver *n*. $I_{n,m}^{\left(t\right)}$ is the set based on the descending order of interference intensity received at the receiver n.

Similarly, the interfered neighborhood set of link *n* on the channel *m* is the set of the receivers interfered by the transmitter *n* at the previous time slot t−1. The interfered neighborhood set of link *n* on the channel *m* is denoted as $O_{n,m}^{\left(t\right)}$. In the same way, in order to prioritize the nearby receivers that use channel *m*, those receivers are divided into two groups. The interference intensity received at those receivers interfered by the transmitter *n* (i.e., $h_{n\to j,m}^{\left(t-1\right)}$) is sorted by descending order in order to prioritize the receivers who are severely interfered by the transmitter *n*. $O_{n,m}^{\left(t\right)}$ is the set based on the descending order of interference intensity received at those receivers interfered by the transmitter *n*.

For the later simulation comparisons, the local state information collected by agent *n* on the channel *m* is performed in the same way as [15], and is composed by the following three parts. The first part is the local state information from communication information between the transmitter *n* and the receiver *n* on the channel *m*, which is denoted as $\kappa_{n,m}$, as shown below.

(11) $$\kappa_{n,m}=\left(\alpha_{n,m}^{\left(t-1\right)}p_{n}^{\left(t-1\right)},C_{n}^{\left(t-1\right)},\mu_{n,m}^{\left(t\right)},h_{n\to n,m}^{\left(t\right)},\sum_{l\neq n}\alpha_{l,m}^{\left(t-1\right)}h_{l\to n,m}^{\left(t\right)}p_{l}^{\left(t-1\right)}\right)$$

where $\mu_{n,m}^{\left(t\right)}=h_{n\to n,m}^{\left(t\right)}/\sum_{l\neq n}\alpha_{l,m}^{\left(t-1\right)}h_{l\to n,m}^{\left(t\right)}p_{l}^{\left(t-1\right)}$.

The second part is the local state information from communication information between the receiver *n* and the interfering neighbors in $I_{n,m}^{\left(t\right)}$ on the channel *m*. In order to reflect the distributed method, *c* elements are indexed according to the interference intensity as the second part information, which is denoted as $\nu_{n,m}$, where $\nu_{n,m}$ follows the priority standard of $I_{n,m}^{\left(t\right)}$.

(12) $$\nu_{n,m}=\left(\alpha_{i,m}^{\left(t-1\right)}p_{i}^{\left(t-1\right)},C_{i}^{\left(t-1\right)},h_{i\to n,m}^{\left(t\right)},\mu_{i,m}^{\left(t-1\right)}|\forall i\in I_{n,m}^{\left(t\right)}\right)$$

The third part is the local state information from communication information between the transmitter *n* and the interfered neighbors in $O_{n,m}^{\left(t\right)}$ on the channel *m*. In order to reflect the distributed method, *c* elements are indexed according to the interference intensity as the second part information, which is denoted as $\eta_{n,m}$, where $\eta_{n,m}$ follows the priority standard of $O_{n,m}^{\left(t\right)}$.

(13) $$\eta_{n,m}=\left(h_{n\to j,m}^{\left(t-1\right)},h_{j\to j,m}^{\left(t-1\right)},C_{j}^{\left(t-1\right)},\mu_{j,m}^{\left(t-1\right)},\sum_{l\neq n}\alpha_{l,m}^{\left(t-1\right)}h_{l\to j,m}^{\left(t-1\right)}p_{l}^{\left(t-1\right)}|\forall j\in O_{n,m}^{\left(t\right)}\right)$$

Based on the above three parts of the local state information, the local state information set collected by agent *n* on the channel *m* can be expressed as $s_{n,m}^{\left(t\right)}$, and is shown as follows.

(14) $$s_{n,m}^{\left(t\right)}=\left\{\kappa_{n,m},\nu_{n,m},\eta_{n,m}\right\}$$

Then, the local state information set collected by agent *n* on all the channels is denoted by $s_{n}^{\left(t\right)}=\left\{s_{n,1}^{\left(t\right)},\ldots ,s_{n,M}^{\left(t\right)}\right\}$.

### 4.4. Proposed Framework for Resource Allocation

**Figure 3:**
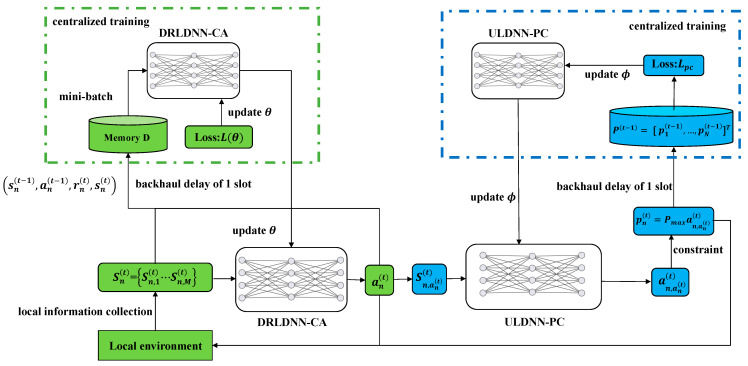
Proposed framework for the resource allocation in P1.

Our algorithm is proposed with *N* agents corresponding to *N* links in the D2D network, rather than just a single agent that controls all the *N* links, because a single learning agent easily suffers the similar drawbacks as centralized execution. The advantage of the proposed algorithm with *N* agents is that only the local state information is obtained for channel selection and power control. In our proposed algorithm, the transmitter *n* is considered as the agent *n*. In addition, each agent in our proposed algorithm executes in a distributed manner with two different deep neural networks, i.e., a deep reinforcement learning-based deep neural network for channel allocation (DRLDNN-CA) and an unsupervised learning-based deep neural network for power control (ULDNN-PC). That is, in the process of distributed executions, each agent can leverage the DRLDNN-CA and the ULDNN-PC to output the channel allocation scheme and the channel power, respectively. Specifically, at each time slot *t*, the DRLDNN-CA in the agent *n* uses the local state information set $s_{n}^{\left(t\right)}$ collected by agent *n* on all the channels as input, and outputs the channel allocation action $a_{n}^{\left(t\right)}$, while the ULDNN-PC in the agent *n* uses the local state information set $s_{n,a_{n}^{\left(t\right)}}^{\left(t\right)}$ collected by agent *n* on the channel $a_{n}^{\left(t\right)}$ as input, and outputs the channel power $p_{n}^{\left(t\right)}$ by constraints. It is noted that both the proposed DRLDNN-CA and the proposed ULDNN-PC are trained in a centralized manner for steadily maximizing the transmit sum-rate. The proposed framework for the resource allocation in P1 is shown in Figure 3.

It can be seen from Figure 3 that the constrained optimization problem P1 is decomposed by the DRLDNN-CA and ULDNN-PC. The channel allocation problem to be solved by the DRLDNN-CA can be described as P2, as expressed below.

(15) $$\begin{array}{l} P2:\underset{\alpha^{\left(t\right)},p^{\left(t\right)}=p^{*(t)}}{\max}\sum_{n=1}^{N}C_{n}^{\left(t\right)}\\ s.t.\;C_{2}:\alpha_{n,m}^{\left(t\right)}\in \left\{0,1\right\},\;\forall n\in \{1,\ldots ,N\},\forall m\in \{1,\ldots ,M\}\\ \;\;\;\;C_{3}:\sum_{m\in M}\alpha_{n,m}^{\left(t\right)}=1,\;\forall n\in \{1,\ldots ,N\} \end{array}$$

where the optimized power allocation scheme $p^{*(t)}$ is provided by the ULDNN-PC network. Similarly, the power allocation problem to be solved by the ULDNN-PC can be described as P3, as expressed below.

(16) $$\begin{array}{l} P3:\underset{\alpha^{\left(t\right)}=\alpha^{*(t)},p^{\left(t\right)}}{\max}\sum_{n=1}^{N}C_{n}^{\left(t\right)}\\ s.t.C_{1}:0\leq p_{n}^{\left(t\right)}\leq P_{\max},\forall n\in \{1,\ldots ,N\} \end{array}$$

where the optimized channel allocation scheme $\alpha^{*(t)}$ is provided by the DRLDNN-CA network.

In the following, both the DRLDNN-CA and the ULDNN-PC are described in detail.

#### 4.4.1. DRLDNN-CA

Based on the constraint optimization problem P2, we define DRLDNN-CA as a channel allocation neural network model, which consists of an input layer, multiple fully connected hidden layers, and an output layer. Each fully connected hidden layer includes a fully connected layer, a batch normalization layer, and a RELU activation function. The output layer of the channel allocation neural network model is regarded as the adaptation value of a link on all channels, and the channel corresponding to the maximum adaptation value is selected as the channel allocation scheme of the link. The DRLDNN-CA can be described as follows:

(1) Input layer: For the agent *n*, the DRLDNN-CA requires the local state information from all channels collected by the agent *n*. That is, the DRLDNN-CA of the agent *n* uses the local state information $s_{n}^{\left(t\right)}$ as its input. According to the local information, the tensor dimension of $s_{n}^{\left(t\right)}$ is M×Q(Q=9×c+5), where *M* is the number of channels, and *c* is the index of the local information.

(2) Output layer: The DRLDNN-CA of the agent *n* uses the discrete action space $a_{n}^{\left(t\right)}\in \{1,\ldots ,M\}$ as its output. That is, the channel $a_{n}^{\left(t\right)}$ is selected by the agent *n* for its transmission, which is then transformed to the solution of the constraint optimization problem P2 by the following rule: $\alpha_{n,m}^{\left(t\right)}=1$ if $m=a_{n}^{\left(t\right)}$, and $\alpha_{n,m}^{\left(t\right)}=0$ if $m\neq a_{n}^{\left(t\right)}$.

(3) Network update: In the DRLDNN-CA network, forward propagation is performed to output the Q value $Q(s_{n}^{\left(t\right)},a,\theta )$. According to the loss function (10) defined by the mean-square Bellman error, the Q value $Q(s_{n}^{\left(t\right)},a,\theta )$ output by the DRLDNN-CA is gradually converged to the target Q value $Q_{target}(s_{n}^{\left(t\right)},a,\theta^{\prime })$. As the DRLDNN-CA is convergent, the optimal policy $\pi^{*}(s)=\max Q(s_{n}^{\left(t\right)},a,\theta^{*})$ is obtained. Then the old weights θ of the DRLDNN-CA are updates by the new weights $\theta^{*}$, and the agent, with the aid of the DRLDNN-CA, generates new actions to interact with the environment.

(4) Reward function: To maximize the problem P1, the reward function of the agent *n* at time slot *t* is set to be $r_{n}^{\left(t\right)}$, and is shown as follows.

(17) $$r_{n}^{\left(t\right)}=C_{n}^{\left(t-1\right)}-\sum_{j\in O_{n,m}^{\left(t\right)}}I_{n\to j}^{\left(t-1\right)}$$

where the reward function consists of two parts, i.e., the direct transmission rate $C_{n}^{\left(t-1\right)}$ of agent *n* and the penalty term $I_{n\to j}^{\left(t-1\right)}=\log\left(1+\frac{\alpha_{j,m}^{\left(t-1\right)}h_{j\to j,m}^{\left(t-1\right)}p_{j}^{*(t-1)}}{\sum_{l\neq n}\alpha_{l,m}^{\left(t-1\right)}h_{l\to j,m}^{\left(t-1\right)}p_{l}^{*(t-1)}+\sigma^{2}}\right)-C_{j}^{\left(t-1\right)}$, $j\in O_{n,m}^{\left(t\right)}$. Note that $C_{n}^{\left(t-1\right)}$ equals to $C_{n,a_{n}^{\left(t\right)}}^{\left(t-1\right)}$, and the power allocation scheme $p^{*(t-1)}$ comes from the ULDNN-PC network. The penalty term reflects, to some extent, the interference strength of the transmitter *n* to all of its interfered neighbors. Obviously, the reward function $r_{n}^{\left(t\right)}$ becomes large when the direct transmission rate $C_{n}^{\left(t-1\right)}$ is large and the penalty term $I_{n\to j}^{\left(t-1\right)}$ is simultaneously small.

(5) Centralized training: To ensure that the distributed execution is more efficient, the centralized training is set up in the DRLDNN-CA network. As shown in Figure 3, an experience pool memory *D* is used. Due to the backhaul delay of one time slot, the latest experience of memory *D* at time slot *t* is $M^{\left(t\right)}=\left\{M_{1}^{\left(t\right)},\ldots ,M_{n}^{\left(t\right)},\ldots ,M_{N}^{\left(t\right)}\right\}$, where $M_{n}^{\left(t\right)}=\left(s_{n}^{\left(t-1\right)},a_{n}^{\left(t-1\right)},r_{n}^{\left(t\right)},s_{n}^{\left(t\right)}\right)$.

In the training of the proposed DRLDNN-CA for the agent *n*, the DQN neural network and target DQN neural network are first created. In addition, memory *D* is redeployed and the relevant parameters, including exploration rate and the DRLDNN-CA, are initialized. The execution of the proposed DRLDNN-CA for the agent *n* is described in Algorithm 1, shown as follows.
**Algorithm 1**: The proposed DRLDNN-CA for the agent *n***input**: Local state information $s_{n,m}^{\left(t\right)}$ from (14)**output**: Channel indexes $a_{n}^{\left(t\right)}$, $a_{n}^{\left(t\right)}\in \{1,\ldots ,M\}$1: Create a DQN with weights *θ* and a target DQN with weights *θ*′ respectively. Initialize *θ* randomly, let *θ*′ = *θ* and *t* = 02: Empty memory *D*3: Set restart experience interval *e*, updating *η* and exploring *ε*4: **repeat**5:   **if** *t* % *e* and *t* ≠ 0      Restart memory *D*6:   **if** rand < *ε*7:       Randomly select an action $a_{n}^{\left(t\right)}$
8:   **else**9:       Generate the action $a_{n}^{\left(t\right)}=\max_{a}Q(s_{n,m}^{\left(t\right)},a,\theta )$ with the DQN10: Update the memory *D* by adding $M^{\left(t\right)}=\left\{M_{1}^{\left(t\right)},\ldots ,M_{n}^{\left(t\right)},\ldots ,M_{N}^{\left(t\right)}\right\}$, where $M_{n}^{\left(t\right)}=\left(s_{n}^{\left(t-1\right)},a_{n}^{\left(t-1\right)},r_{n}^{\left(t\right)},s_{n}^{\left(t\right)}\right)$
11:   **if**
*t* mod *η* = 0 **then**12:       Randomly sample a batch of data set $M^{\left(t\right)}$ from memory *D*13:       Centralized Training: Train the DQN with $M^{\left(t\right)}$ and minimize the loss function (10) to update *θ*14: Let *t* = *t* + 115: **until** *t* > $t_{\max}$
16:   **end**17: **end**

#### 4.4.2. ULDNN-PC

Based on constraint optimization problem P3, the ULDNN-PC is defined as a power control neural network model, which consists of one input layer, multiple fully connected hidden layers, one output layer, and one constraint layer. Each fully connected hidden layer includes a fully connected layer, a batch normalization layer, and a RELU activation function, where the output constraint layer processes the output of the output layer to satisfy the constraint. The ULDNN-PC can be described as follows:

(1) Input layer: In this paper, the ULDNN-PC network is constructed from the constraint problem P3, which is a fully connected deep forward propagation network. The ULDNN-PC network uses the local state information $s_{n,a_{n}^{\left(t\right)}}^{\left(t\right)}$ as its input, where $a_{n}^{\left(t\right)}\in \{1,2,\ldots ,M\}$ is the channel allocation scheme obtained by the DRLDNN-CA and the tensor dimension of $s_{n,a_{n}^{\left(t\right)}}^{\left(t\right)}$ is Q(Q=9×c+5), and *c* is the index of the local information. Note that $s_{n,a_{n}^{\left(t\right)}}^{\left(t\right)}\subset \left\{s_{n,1}^{\left(t\right)},s_{n,2}^{\left(t\right)},\ldots ,s_{n,M}^{\left(t\right)}\right\}$.

(2) Output layer: In the ULDNN-PC, the fully connected hidden layers sequentially consist of one fully connected layer, one normalized layer, and one ReLU activation layer. In order to meet the continuous nature of the power, the activation function of the output layer of the ULDNN-PC is defined as the Sigmoid function. That is, the output of the ULDNN-PC $a_{n,a_{n}^{\left(t\right)}}^{\left(t\right)}$ satisfies $a_{n,a_{n}^{\left(t\right)}}^{\left(t\right)}\in (0,1)$. In order to make the output of the ULDNN-PC satisfy the constrained optimization problem P3, the following constraint (18) is performed.

(18) $$p_{n}^{\left(t\right)}=p_{\max}\cdot a_{n,a_{n}^{\left(t\right)}}^{\left(t\right)}$$

(3) Centralized training: The constraint optimization problem P3 is modeled and analyzed by the unsupervised learning strategy, where the label data are not required. The loss function used in the unsupervised learning strategy for centralized training is defined as follows.

(19) $$L_{pc}=-E_{p^{\left(t-1\right)}}\left(\sum_{n=1}^{N}{\bar{C}}_{n}^{\left(t-1\right)}\right)$$

where ${\bar{C}}_{n}^{\left(t-1\right)}$ is the transmit sum-rate with gradient information, which allows for the performance of back propagation of the neural network, and $E_{x}\left(\cdot \right)$ represents the operation of expectations on *x*. Note that ${\bar{C}}_{n}^{\left(t-1\right)}$ can be obtained from locally known information in the computational environment, and the value of ${\bar{C}}_{n}^{\left(t-1\right)}$ is equal to that of $C_{n}^{\left(t\right)}$.

The execution of the proposed ULDNN-PC for the agent *n* is described in Algorithm 2, which is shown as follows.
**Algorithm 2**: The proposed ULDNN-PC for the agent *n***input**: Local state information $s_{n,a_{n}^{\left(t\right)}}^{\left(t\right)}$ determined by the DRLDNN-CA**output**: $a_{n,a_{n}^{\left(t\right)}}^{\left(t\right)}$(before the constraints)1: Create a DNN with weights θ and initialize θ
2: Set *t* = 03: **repeat**4:   Generate the output action $a_{n,a_{n}^{\left(t\right)}}^{\left(t\right)}\in (0,1)$ with the DNN 5:   $p_{n}^{\left(t\right)}=p_{\max}\cdot a_{n,a_{n}^{\left(t\right)}}^{\left(t\right)}$ (after the constraints)6:   **if** agent = (*N* − 1) **then**7:    Centralized Training: Train the DNN with $L_{pc}$ and minimize the loss function (19) to update θ
8:   Let *t* = *t* + 19: **until** $t>t_{\max}$
10:   **end**11: **end**

#### 4.4.3. Methodology of the DRLDNN-CA and ULDNN-PC

**Figure 4:**
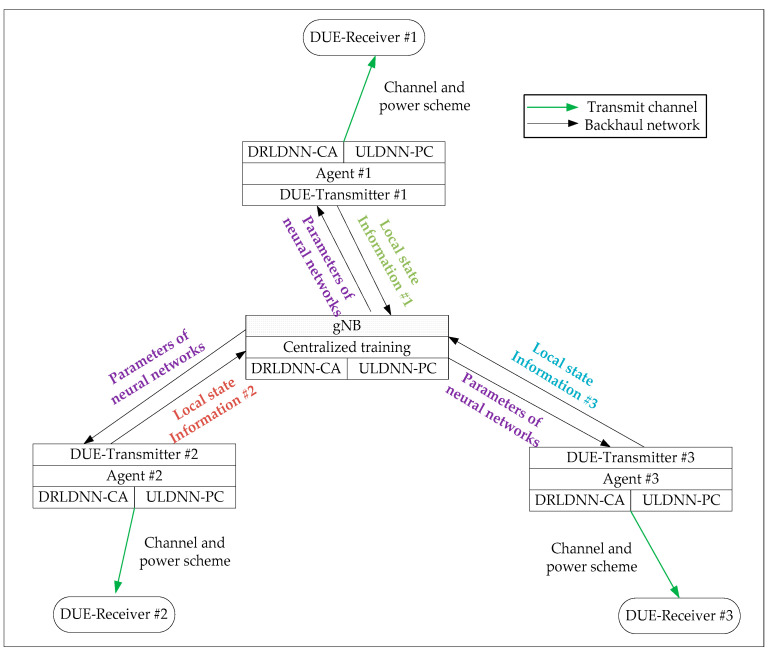
Schematic diagram of application of the proposed joint of the DRLDNN-CA and the ULDNN-PC to DUEs of D2D wireless networks in 5G.

In this section, we make more explanations on the application of our proposed algorithm, i.e., a joint of the DRLDNN-CA and ULDNN-PC, to DUEs of D2D wireless networks in 5G. For this end, we provide a schematic diagram shown in Figure 4. As shown in Figure 4, there are three pairs of D2D transceivers (DUEs) and one 5G base station (i.e., gNB). The base station of 5G (gNB) is responsible for the centralized training of both the DRLDNN-CA and the ULDNN-PC by using the collected local state information from the D2D DUEs. As both the DRLDNN-CA and the ULDNN-PC are trained once, the parameters of both the DRLDNN-CA and the ULDNN-PC are transmitted to the transmitters of the DUEs. This means that all the transmitters of the DUEs share the same parameters of the neural networks. Based on different local state information, the transmitters of the DUEs can decide their channel and power schemes.

As opposed to other methods [14,15,16,17,18,21,22,23,24,25,26] for the resource allocation of D2D wireless networks, our proposed framework with a joint of the DRLDNN-CA and the ULDNN-PC is distributed for solving the resource allocation. Especially, it is based on the unsupervised learning mechanism that the ULDNN-PC uses for the power control. Note that, concerning the base station of 5G (gNB), the centralized training for the ULDNN-PC is easily completed, because the whole gradient information for the ULDNN-PC can be stored in the base station and can be used to calculate the gradient descent conveniently based on the outputs of the neural network.

Note that transmissions of the parameters of neural networks and the local information through the backhaul network have to take certain time slots. This means that there exist a certain number of time delays in the updates of the parameters of neural networks and the local state information. However, the negative effects of the time delays can be mitigated by the neural networks to enable nonlinear mapping [15]. In simulations, we consider the time delays as the backhaul delay of one time slot.

It is worth mentioning that our proposed algorithm uses the optimized schemes of both channel and power to mitigate channel interference among the DUEs of D2D wireless networks. To be specific, the discrete channel variable and the continuous power variable are optimized by the joint of the DRLDNN-CA and the ULDNN-PC to maximize the system sum-rate rates, thereby mitigating the channel interference. In the optimization process, the ULDNN-PC is used to generate the optimized power to maximize the system sum-rate rates, while the DRLDNN-CA, by combining the optimized power generated by the ULDNN-PC, is used to generate the optimized channel to maximize the reward function (17). It can also be seen from Equation (17) that the reward function can be maximized by minimizing the channel interference among DUEs. However, it is difficult for our proposed algorithm framework to be applied to mitigate the channel interference among cellular users (CUs) and gNBs directly. Both the reward function of DQN and the loss functions need to be designed elaborately to mitigate the channel interference among CUs and gNBs.

## 5. Simulation Results

In this section, the proposed algorithm in this paper is compared with the traditional optimization algorithms and the deep reinforcement learning algorithms.

**Figure 5:**
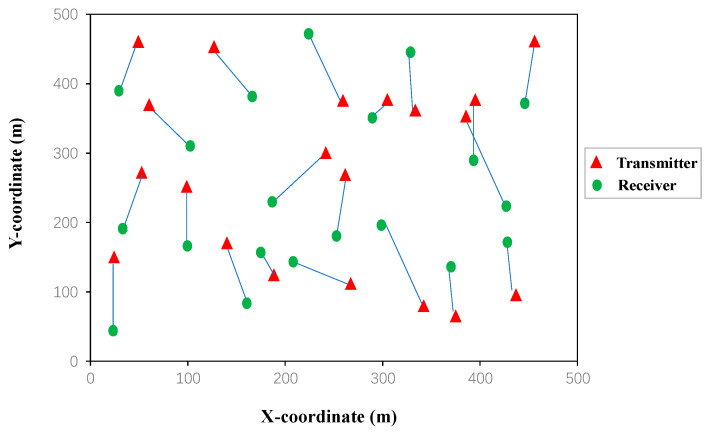
The topology of the D2D network with 20 links.

According to the LTE standard [30], the path loss is given by 128.1 + 37.6 log_10_(*d*) (in dB), where *d* denotes the distance between a transmitter and a receiver in km. We set *P*_max_ = 38 dBm and *σ*^2^ = −114 dBm. Similar to [31], due to the practical front-end dynamic range limitation, we set the upper limit of the signal-to-noise ratio to 30 dBm. In simulations, we chose four D2D networks with different (links, channels), i.e., (20 links, 2 channels), (20 links, 4 channels), (50 links, 5 channels), and (50 links, 10 channels). The topology of a D2D network with 20 links used in the simulation is plotted in Figure 5, where each D2D transmitter pair was randomly placed on a plain with an area of 500 × 500 m, and each receiver was randomly placed around its transmitter between 10 m and 100 m. For the sake of fairness, training parameters of all the benchmark algorithms had the same order of the magnitude, and all the offline training were performed under the same hardware configurations with NVIDIA GeForce RTX 3060 6G GPU, 12th Gen Intel(R) Core(TM) i7-12700H 2.70 GHz and 16G RAM. What is more, only the CPU was used for the online inference of all benchmark algorithms and performance comparisons among different algorithms.

**Figure 6:**
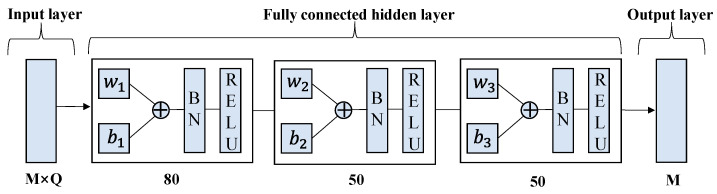
Structure of DRLDNN-CA.

**Figure 7:**
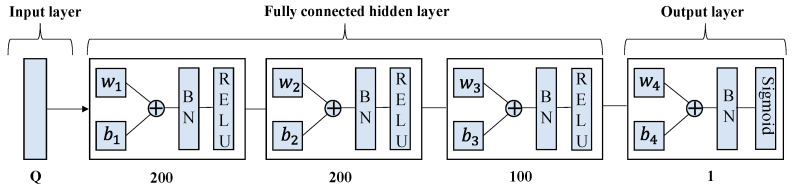
Structure of ULDNN-PC.

In order to prevent gradient explosion, we added the Batch Normalization (BN) layer to the network and determined the DRLDNN-CA and ULDNN-PC structures through trial and error as shown in Figure 6 and Figure 7, respectively. The learning rate was determined as 0.001 and 0.0001, respectively. The DRLDNN-CA input layer dimension was *M* × *Q*, *Q* = 9 × *c* + 5, *c* = 5, the respective dimensions of the fully connected hidden layer from left to right were 80, 50 and 50, and the output layer dimension was *M*, where *M* is the number of channels. The ULDNN-PC input layer dimension was *Q*, *Q* = 9 × *c* + 5, *c* = 5, the respective dimensions of the fully connected hidden layer from left to right were 200, 200, and 100, and the output layer dimension was 1.

To illustrate the proposed distributed method for the channel allocation and power control in detail, we set up a simple example where the number of channels was 2 and the number of links was 20. It meant that there were 20 agents corresponding to 20 links in the proposed distributed method, and each agent contained both the DRLDNN-CA and the DRLDNN-PC. According to the local information $s_{n}^{\left(t\right)}=\left\{s_{n,1}^{\left(t\right)},\ldots ,s_{n,M}^{\left(t\right)}\right\}$ (*n* = 1,2,...,20, *M* = 2), the local information of the 20 links could be described as $s_{1}^{\left(t\right)}=\left\{s_{1,1}^{\left(t\right)},s_{1,2}^{\left(t\right)}\right\}$, $s_{2}^{\left(t\right)}=\left\{s_{2,1}^{\left(t\right)},s_{2,2}^{\left(t\right)}\right\}$, …, $s_{20}^{\left(t\right)}=\left\{s_{20,1}^{\left(t\right)},s_{20,2}^{\left(t\right)}\right\}$. In addition, the DRLDNN-CA of the agent *n* took $s_{n}^{\left(t\right)}$ as its input, while the DRLDNN-PC of the agent *n* took one of $s_{n,1}^{\left(t\right)},\ldots ,s_{n,M}^{\left(t\right)}$ as its input. Note that the input of the DRLDNN-PC of the agent was determined by the output of the DRLDNN-CA.

In the following, we took the agent 1 as an example, where the local information of the link 1 were used by both the DRLDNN-CA and the DRLDNN-PC of the agent 1 to obtain the channel allocation scheme and the power allocation scheme. According to the local information set described in Section 4.3, the dimension of all the local information is *M* × *Q*, where *M* = 2, *Q =* 9 × *c +* 5, and *c* is the number of interference indexes of the local information set. Assume *c =* 5. Then, the local information $s_{1}^{\left(t\right)}$ contains 100 elements, $s_{1,1}^{\left(t\right)}$ contains 50 elements, and $s_{1,2}^{\left(t\right)}$ contains 50 elements, which are shown as follows.

(20) $$s_{1,1}^{\left(t\right)}=\left[\begin{array}{ccccc} 0.\;, & -1.22838546, & 0.06711202, & -1.\;, & 0.\;,\\ 1.9003183, & 1.9003183\;, & 1.9003183\;, & 1.9003183\;, & 0.24436685\;,\\ \;\cdots ,\;\cdots , & \cdots ,\; & \cdots ,\;\cdots , & \cdots ,\;\cdots , & \cdots ,\\ \;0.21980716, & \;0.1375979\;, & \;0.58749212\;, & \;0.34550899,\; & 0.7405233\;,\\ -0.94919191,\; & -0.96879865\;, & \;-0.99465654,\; & -0.91036299,\; & -0.36410471,\; \end{array}\right]$$

(21) $$s_{1,2}^{\left(t\right)}=\left[\begin{array}{ccccc} \;1.7218569\;, & \;2.12528973,\; & -0.89313561,\; & -0.73803674, & -0.42873594,\\ \;3.68788099, & \;4.09131381, & \;1.79593686\;, & \;1.65594349\;, & \;1.40660372\;,\\ \;\cdots ,\;\cdots , & \cdots ,\; & \cdots ,\;\cdots , & \cdots ,\;\cdots , & \cdots ,\\ \;1.73109764, & \;2.18301908, & \;0.1232268\;, & \;0.03551651\;, & \;0.04313387\;,\\ -0.90344739,\; & -0.93558354,\; & -0.98836712, & \;-0.96701174\;, & \;-0.92352049 \end{array}\right]$$

(22) $$s_{1}^{\left(t\right)}=\left\{s_{1,1}^{\left(t\right)},s_{1,2}^{\left(t\right)}\right\}=\left[\begin{array}{ccccc} \;0.\;, & \;-1.22838546, & \;0.06711202\;, & \;-1.\;, & 0.\;,\\ \;1.9003183\;, & \;1.9003183\;, & \;1.9003183\;, & \;1.9003183\;, & \;0.24436685\;,\\ \;0.21980716,\; & 0.1375979\;, & \;0.58749212\;, & \;0.34550899,\; & 0.7405233\;,\\ -0.94919191,\; & -0.96879865\;, & \;-0.99465654,\; & -0.91036299,\; & -0.36410471,\\ \;\cdots ,\;\cdots , & \cdots ,\; & \cdots ,\;\cdots , & \cdots ,\;\cdots , & \cdots ,\\ \;1.7218569\;, & \;2.12528973,\; & -0.89313561,\; & -0.73803674,\; & -0.42873594,\\ \;3.68788099,\; & 4.09131381,\; & 1.79593686\;, & \;1.65594349\;, & \;1.40660372\;,\\ \;1.73109764,\; & 2.18301908,\; & 0.1232268\;, & \;0.03551651\;, & \;0.04313387\;,\\ -0.90344739,\; & -0.93558354,\; & -0.98836712,\; & -0.96701174\;, & \;-0.92352049 \end{array}\right]$$

Let the local information $s_{1}^{\left(t\right)}=\left\{s_{1,1}^{\left(t\right)},s_{1,2}^{\left(t\right)}\right\}$ as the input of the DRLDNN-CA of the agent 1. Assume that the output of the DRLDNN-CA is shown as (23).

(23) $$output=\left[0.0058,\;0.0353\right]$$

We regarded the output layer of DRLDNN-CA as the adaptation value of a link on all channels, and selected the channel with the maximum adaptation value as the channel allocation scheme of the link. Hence, $\alpha_{1,2}^{\left(t\right)}=1$ and $\alpha_{1,1}^{\left(t\right)}=0$. It meant that the channel allocation scheme could be expressed as $a_{1}^{\left(t\right)}=2$.

The dimension of the input of the ULDNN-PC was *Q*, *Q* = 9 *× c* + 5. That is, the dimension of the input layer of ULDNN-PC was 50. Note that the ULDNN-PC of the agent 1 used a set of the local information determined by the output channel allocation scheme of the DRLDNN-CA of the agent 1 as input. Based on the above, the input layer of DRLDNN-CA is $s_{1,a_{1}^{\left(t\right)}}^{\left(t\right)}$, which is denoted as:

(24) $$s_{1,a_{1}^{\left(t\right)}}^{\left(t\right)}=s_{1,2}^{\left(t\right)}=\left[\begin{array}{ccccc} 1.7218569\;, & \;2.12528973, & \;-0.89313561,\; & -0.73803674,\; & -0.42873594,\\ \;3.68788099,\; & 4.09131381,\; & 1.79593686\;, & \;1.65594349\;,\; & 1.40660372\;,\\ \;\cdots ,\;\cdots , & \cdots ,\; & \cdots ,\;\cdots , & \cdots ,\;\cdots , & \cdots ,\\ 1.73109764,\; & 2.18301908,\; & 0.1232268\;, & \;0.03551651\;,\; & 0.04313387\;,\\ -0.90344739,\; & -0.93558354,\; & -0.98836712,\; & -0.96701174\;,\; & -0.92352049\; \end{array}\right]$$

Assume that the output of the ULDNN-PC is expressed as (25). In order to meet the constraints in this paper, we take $p_{1}^{\left(t\right)}=p_{\max}\cdot a_{1,a_{1}^{\left(t\right)}}^{\left(t\right)}$, where *P*_max_ = 38 dBm. Then, the output power of the ULDNN-PC is 3.289 W.

(25) $$a_{1,a_{1}^{\left(t\right)}}=a_{1,2}=\left[0.5203\right]$$

Our proposed algorithm was used to compare with five benchmark algorithms in this paper. For convenience, our proposed algorithm is denoted as ‘Proposed DRLDNN-CA + ULDNN-PC’. The first benchmark algorithm is called the ‘Joint DQN + DDPG’ as proposed in [15]. The second is called the ‘Joint DQN’ as proposed in [14], where the transmit power is discretized into 10 levels for comparisons. The third and the fourth are different variants from the optimal fractional planning (FP) algorithm [8]. The third is called the ‘Ideal FP’, which requires a fully real-time CSI to run the fractional planning algorithm and ignores the delay between performing the centralized optimization and passing the optimization results to the transmitter. The fourth is called the ‘Delayed FP’, which indicates a delay of one time slot to run the fractional planning algorithm. The last is the random allocation algorithm, called the ‘Random’.

In the training phase, four episodes with each running for 5000 time slots were used to train our proposed DRLDNN-CA and ULDNN-PC. In order to speed up the convergence rate, the memory *D* was always reset at the beginning of each episode.

**Figure 8:**
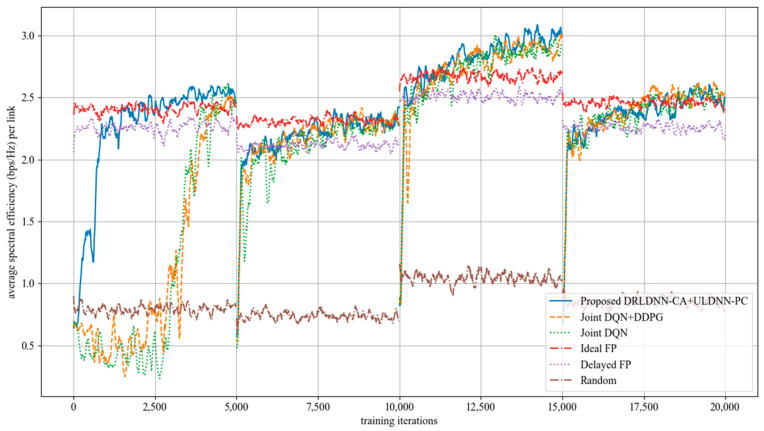
Average transmit sum-rate with 20 links and 2 channels (*N* = 20, *M* = 2).

**Figure 9:**
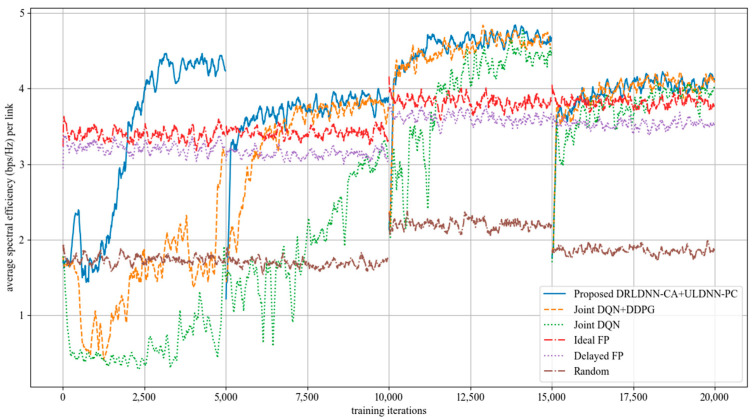
Average transmit sum-rate with 20 links and 4 channels (*N* = 20, *M* = 4).

The average transmit sum-rate for 20 links and 2 channels (*N* = 20, *M* = 2) is shown in Figure 8, while that for 20 links and 4 channels (*N* = 20, *M* =4) is shown in Figure 9. As seen from Figure 8 and Figure 9, within the training of the first episode, our proposed algorithm was superior to both the Joint DQN [14] and the Joint DQN + DDPG [15] in the convergent rate and the average transmit sum-rate. It also can be seen from comparisons between Figure 8 and Figure 9 that the advantage of our proposed algorithm over other algorithms became more and more obvious. For example, within the training of the second episode, our proposed algorithm was close to both the Joint DQN and the Joint DQN + DDPG in Figure 8 with 20 links and 2 channels, while our proposed algorithm was superior to both the Joint DQN and the Joint DQN + DDPG in Figure 9 with 20 links and 4 channels.

**Figure 10:**
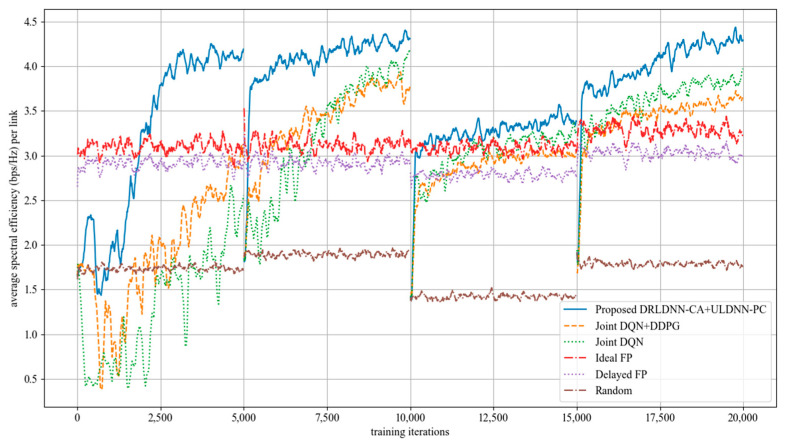
Average transmit sum-rate with 50 links and 5 channels (*N* = 50, *M* = 5).

**Figure 11:**
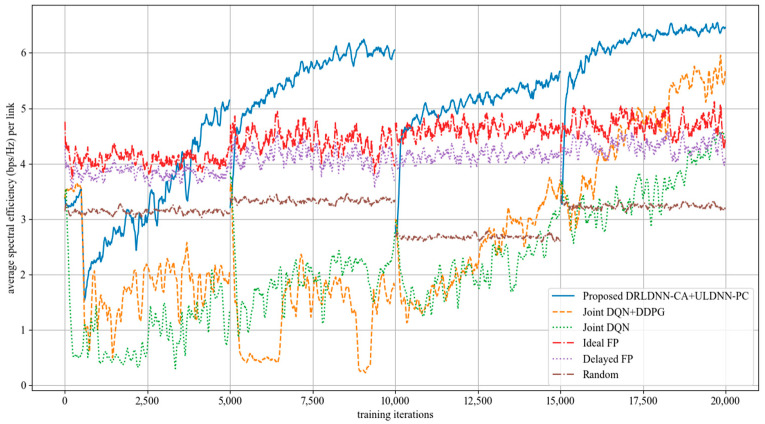
Average transmit sum-rate with 50 links and 10 channels (*N* = 50, *M* = 10).

The average transmit sum-rate for 50 links and 5 channels (*N* = 50, *M* = 5) is shown in Figure 10, while that for 50 links and 10 channels (*N* = 50, *M* =10) is shown in Figure 11. It also can be seen from Figure 10 and Figure 11 that our proposed algorithm was superior to other algorithms with the increasing of the channel (*M*) at 50 links. The gap in the obtained transmit sum-rate became more and more obvious as both the links and the channels increased, which indicates that our proposed algorithm can obtain larger average transmit sum-rates than other algorithms at larger links and channels.

The train results of the average of sum-rate performance and the train results of the variance of sum-rate performance are summarized in Table 2 and Table 3. The results in Table 2 show that our proposed algorithm could obtain larger average transmit sum-rate than other benchmark algorithms, which suggests that our proposed algorithm has better scalability than other benchmark algorithms. The results in Table 3 show that the multi-channel and multi-link transmit sum-rate of our proposed method was stable and efficient.

**Figure 12:**
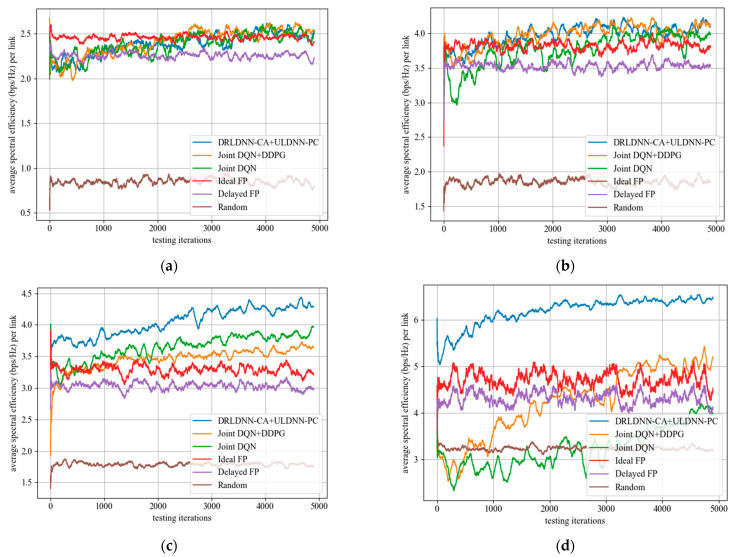
Average transmit sum-rate during the test phase. (**a**) *M* = 2 channels, *N* = 20 links; (**b**) *M* = 4 channels, *N* = 20 links; (**c**) *M* = 5 channels, *N* = 50 links; (**d**) *M* = 10 channels, *N* = 50 links.

When the training was finished, all the algorithms with their trained policies were used in the test phase for further comparisons. In the test phase, deployments were randomly generated, which were different from those used in the training phase. In order to get the test results more quickly, we set one episode for 5000 times slots. Figure 12 shows the test results of the proposed algorithm and other benchmark algorithms in different channels and different links. It can be seen from the figure that the performance of the algorithm proposed in this paper was relatively stable with the increase in the number of channels and links, and the transmit sum-rates were higher than other benchmark algorithms with the increase in the number of channels and links.

The test results of the average of sum-rate performance and the test results of the variance of sum-rate performance are summarized in Table 4 and Table 5. Results in Table 4 show that our proposed algorithm with the trained policy obtained larger average transmit sum-rates than other benchmark algorithms, which suggests that our proposed algorithm has better scalability than other benchmark algorithms. Results in Table 5 show that the multi-channel and multi-link transmit sum-rate of our proposed method was stable and efficient, and the transmit sum-rate did not degrade with the increase in the number of channels and links.

**Figure 13:**
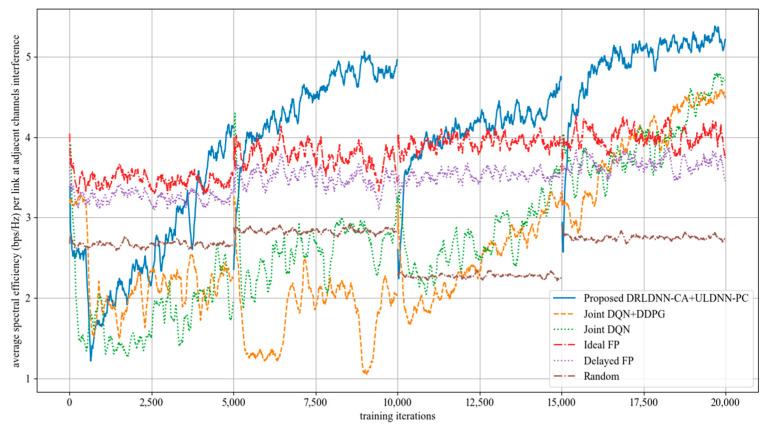
Average transmit sum-rate as the maximum distance of adjacent channels equals 1 (*z* = 1).

**Figure 14:**
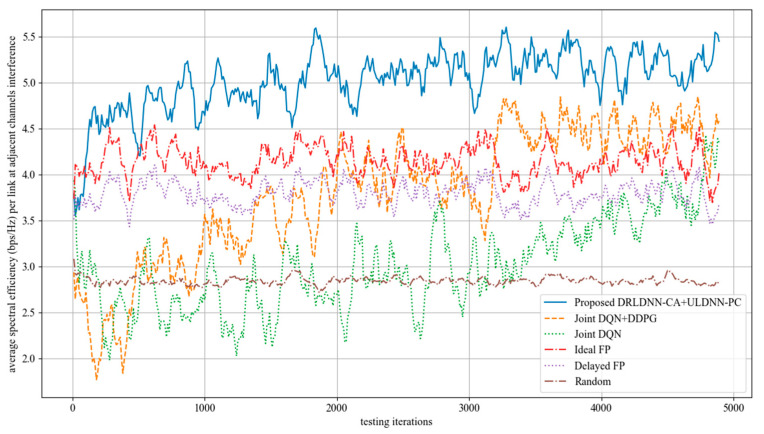
Average transmit sum-rate during test phase as the maximum distance of adjacent channels equals 1 (*z* = 1).

As the channels are not orthogonal, the interference from adjacent channels comes into existence to impair the system sum rate. In the following, we evaluated the performance of our proposed joint algorithm of the DRLDNN-CA and the ULDNN-PC by comparisons with other benchmark algorithms as the interference from adjacent channels occurred. We first made comparisons on the training phase at 50 links and 10 channels as the maximum distance of adjacent channels equal to 1, i.e., *z* = 1. This means that the interference existed among the channels *n*, *n* + 1, and *n* − 1. The simulation results are shown in Figure 13. It can be seen from Figure 13 that the average transmit sum-rates of all the algorithms decayed during the training phase because of the interference from adjacent channels. However, our proposed algorithm was superior to other benchmark algorithms as the training iterations exceeded 5000. After the training phase, the well-trained neural networks were then used for testing. The testing results are shown in Figure 14. The testing results indicate that our proposed joint algorithm of the DRLDNN-CA and the ULDNN-PC outperformed the other benchmark algorithms in the testing phase.

**Figure 15:**
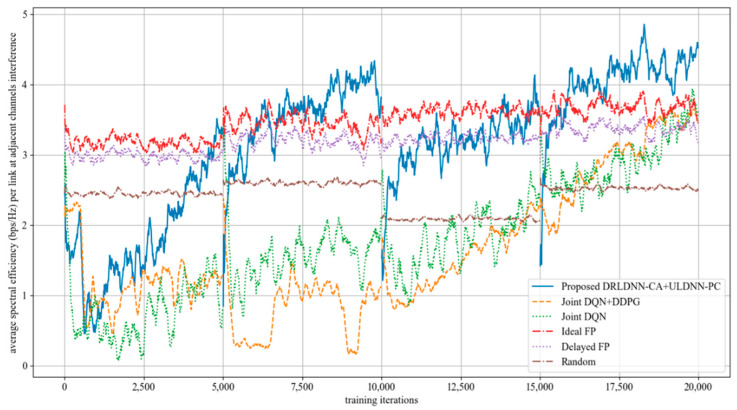
Average transmit sum-rate as the maximum distance of adjacent channels equals 1 (*z* = 2).

**Figure 16:**
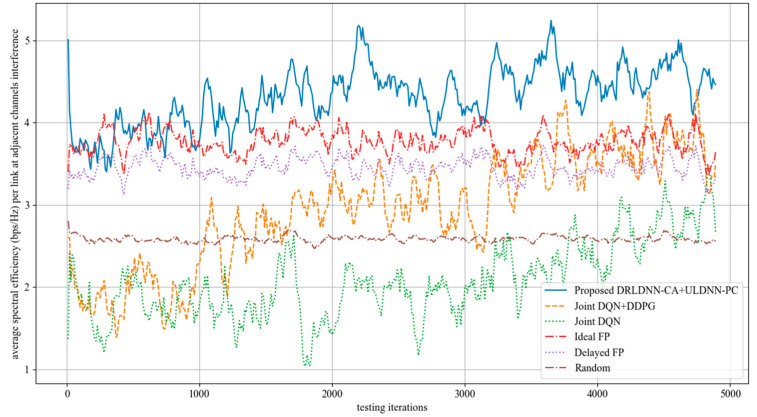
Average transmit sum-rate during test phase as the maximum distance of adjacent channels equals 1 (*z* = 2).

Further, we made comparisons on the training phase at 50 links and 10 channels as the maximum distance of adjacent channels equaled 2, i.e., *z* = 2. This means that the interference existed among the channels *n*, *n* + 1, *n* + 2, *n* − 1, and *n* − 2. The simulation results are shown in Figure 15. It can be seen from Figure 15 that the average transmit sum-rates of all the algorithms decayed more seriously during the training phase because the interference from adjacent channels became increasingly large with the increase in the maximum distance of adjacent channels. However, our proposed algorithm was superior to other benchmark algorithms as the training iterations exceeded 7500. In the same way, the well-trained neural networks were used for testing after the training phase. The testing results are shown in Figure 16. The testing results in Figure 16 indicate that our proposed joint algorithm of the DRLDNN-CA and the ULDNN-PC outperformed the other benchmark algorithms in the testing phase.

The simulation results show that our proposed joint algorithm of the DRLDNN-CA and the ULDNN-PC still works well when the interference from adjacent channels exists in the D2D wireless network.

**Figure 17:**
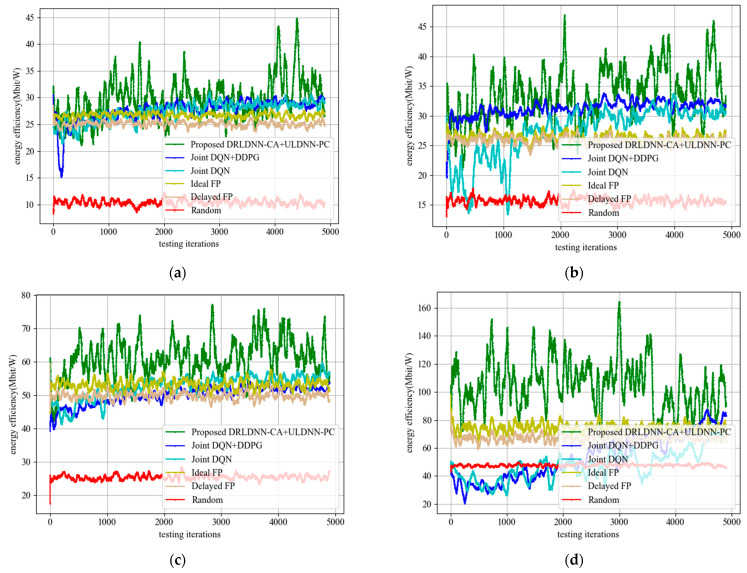
Energy efficiency during the test phase. (**a**) *M* = 2 channels, *N* = 20 links; (**b**) *M* = 4 channels, *N* = 20 links; (**c**) *M* = 5 channels, *N* = 50 links; (**d**) *M* = 10 channels, *N* = 50 links.

Next, we tested the performance of the proposed DRLDNN-CA + ULDNN-PC algorithm in energy efficiency. Figure 17 shows the comparison results between the proposed DRLDNN-CA + ULDNN-PC algorithm and the other benchmark algorithms with the increase in the number of channels and links. It can be seen from Figure 17 that the energy efficiency obtained by the proposed algorithm was higher than the other benchmark algorithms in terms of the number of channels and links.

**Figure 18:**
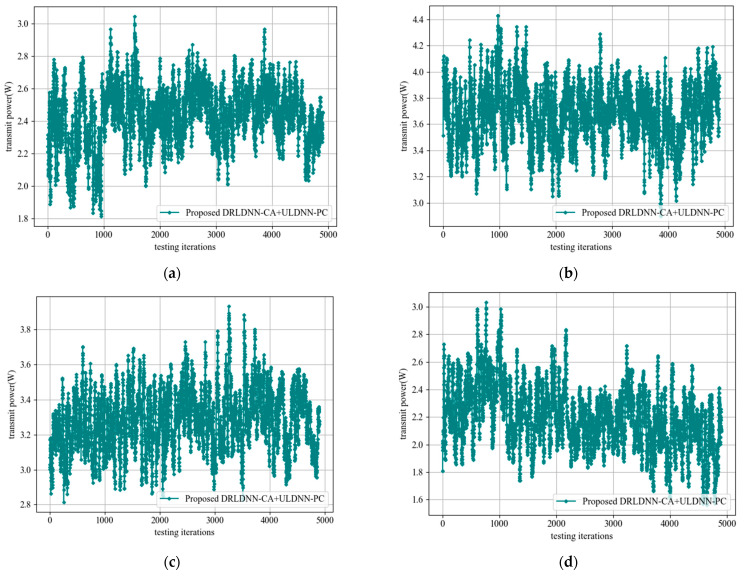
Transmit power during the test phase. (**a**) *M* = 2 channels, *N* = 20 links; (**b**) *M* = 4 channels, *N* = 20 links; (**c**) *M* = 5 channels, *N* = 50 links; (**d**) *M* = 10 channels, *N* = 50 links.

Finally, we considered the legitimacy of transmission power under the constraints presented in this paper. We tested the transmit power obtained by the proposed DRLDNN-CA + ULDNN-PC distributed algorithm and plotted the variation curves of the transmit power, as seen in Figure 18, with the increase in the number of channels and links. It can be seen from Figure 18 that the transmit power obtained by the proposed DRLDNN-CA + ULDNN-PC distributed algorithm was within the power constraint range, which proves the reliability of the proposed algorithm.

## 6. Conclusions

With centralized training and distributed execution, a joint deep Q network and unsupervised learning network, i.e., the joint DRLDNN-CA and ULDNN-PC, has been proposed to address the problem of channel selection and power control in multichannel D2D networks and to maximize sum-rate. With the increase in links and channels in the D2D network, the proposed joint DRLDNN-CA and ULDNN-PC increasingly outperformed other benchmark algorithms. The well-trained joint DRLDNN-CA and ULDNN-PC was more scalable in the average transmit sum-rate for randomly generated deployments than other benchmark algorithms.

## Figures and Tables

**Table 1:** Comparisons between our proposed algorithm and other state-of-the-art algorithms for D2D wireless networks related to 5G.

Publication	Algorithm	Model	Resource Allocation	Approach
Tan et al. [14]	Reinforcement	DQN	Channel select, Power control	Distributed
Lee et al. [16]	Unsupervised	CNN	Power control	Centralized
Lee et al. [26]	Unsupervised	DNN	Power control	Centralized
Nasir et al. [15]	Reinforcement	DQN, DDPG	Channel select, power control	Distributed
Zhang et al. [25]	Supervised	CNN	Power control	Centralized
Yuan et al. [21]	Reinforcement	DQN	Channel select, power control	Distributed
Bi et al. [22]	Reinforcement	DQN	Power control	Centralized
Yu et al. [23]	Reinforcement	DQN	Channel select, power control	Distributed
Lee et al. [17]	Unsupervised	DNN	Power control	Centralized
Chandra et al. [24]	Reinforcement, supervised	DQN, SVM	Power control	Distributed
This paper	Reinforcement, unsupervised	DQN, DNN	Channel select, power control	Distributed

**Table 2:** Train results of average of sum-rate performance.

N Links	M Channels	Proposed	Joint DQN + DDPG	Joint DQN	Ideal FP	Delayed FP	Random
20	2	2.59	2.57	2.55	2.57	2.38	0.95
	4	4.26	4.23	3.85	3.82	3.57	2.03
50	5	3.64	3.16	3.32	3.19	2.91	1.60
	10	5.63	3.56	2.84	4.67	4.23	2.95

**Table 3:** Train results of variance of sum-rate performance.

N Links	M Channels	Proposed	Joint DQN + DDPG	Joint DQN	Ideal FP	Delayed FP	Random
20	2	3.76	3.70	4.02	1.98	2.25	2.68
	4	5.74	5.75	10.14	8.37	6.93	6.16
50	5	8.85	11.38	13.56	17.07	11.55	5.39
	10	26.66	45.04	39.51	31.87	34.73	30.96

**Table 4:** Test results of average of sum-rate performance.

N Links	M Channels	Proposed	Joint DQN + DDPG	Joint DQN	Ideal FP	Delayed FP	Random
20	2	2.41	2.42	2.38	2.46	2.26	0.85
	4	3.98	3.97	3.76	3.82	3.53	1.85
50	5	4.05	3.47	3.65	3.29	3.03	1.79
	10	6.10	4.26	3.24	4.71	4.32	3.23

**Table 5:** Test results of variance of sum-rate performance.

N Links	M Channels	Proposed	Joint DQN + DDPG	Joint DQN	Ideal FP	Delayed FP	Random
20	2	2.07	2.21	2.42	1.36	1.55	2
	4	3.02	3.14	5.20	6.97	5.53	4.64
50	5	4.98	5.73	5.57	5.24	5.09	5.28
	10	8.98	34.30	18.81	12.52	14.32	13.23

## Data Availability

Not applicable.
